# *Osblr8* orchestrates intrachromosomal loop structure required for maintaining stem cell pluripotency

**DOI:** 10.7150/ijbs.45112

**Published:** 2020-04-06

**Authors:** Yanbo Zhu, Zi Yan, Zhonghua Du, Shilin Zhang, Changhao Fu, Ying Meng, Xue Wen, Yizhuo Wang, Andrew R. Hoffman, Ji-Fan Hu, Jiuwei Cui, Wei Li

**Affiliations:** 1 Key Laboratory of Organ Regeneration and Transplantation of Ministry of Education, Stem Cell and Cancer Center, The First Hospital of Jilin University, Changchun, Jilin 130021, China; 2 Division of Endocrinology and Metabolism, The First Hospital of Jilin University, Changchun, Jilin 130021, China; 3 Stanford University Medical School, VA Palo Alto Health Care System, Palo Alto, CA 94304, USA

**Keywords:** lncRNA, pluripotency, stem cell, intrachromosomal interaction, epigenetics

## Abstract

Induced pluripotent stem cells (iPSCs), derived from reprogramming of somatic cells by a cocktail of transcription factors, have the capacity for unlimited self-renewal and the ability to differentiate into all of cell types present in the body. iPSCs may have therapeutic potential in regenerative medicine, replacing injured tissues or even whole organs. In this study, we examine epigenetic factors embedded in the specific 3-dimensional intrachromosomal architecture required for the activation of endogenous pluripotency genes. Using chromatin RNA *in situ* reverse transcription sequencing (CRIST-seq), we identified an *Oct4*-*Sox2* binding long noncoding RNA, referred as to *Osblr8*, that is present in association with pluripotency status. *Osblr8* was highly expressed in iPSCs and E14 embryonic stem cells, but it was silenced in fibroblasts. By using shRNA to knock down *Osblr8*, we found that this lncRNA was required for the maintenance of pluripotency. Overexpression of *Osblr8* activated endogenous stem cell core factor genes. Mechanistically, *Osblr8* participated in the formation of an intrachromosomal looping structure that is required to activate stem cell core factors during reprogramming. In summary, we have demonstrated that lncRNA *Osblr8* is a chromatin architecture modulator of pluripotency-associated master gene promoters, highlighting its critical epigenetic role in reprogramming.

## Introduction

Induced expression of four defined factors that are important for maintaining the pluripotent properties of embryonic stem cells can epigenetically reprogram somatic cells to a pluripotent state, resulting in the generation of induced pluripotent stem cells (iPSCs) 1-3. Unfortunately, the induction of iPSCs from somatic cells using defined factors is an extremely inefficient process 4, 5. In reprogramming, epigenetic factors play a very important role in activating the transcriptional network associated with pluripotency 6, 7. A clear understanding of these cellular factors is very important for improving the efficiency of iPSC induction, allowing for the successful therapeutic application of these pluripotent cells.

In a previous study characterizing epigenetic barriers in reprogramming, we compared the promoter DNA binding and chromatin architecture between iPSCs that completed reprogramming and non-iPSCs that expressed the four defined factors (*Oct4-Sox2-klf4-c-Myc*, OSKM) but failed to complete reprogramming 8. The virally expressed OSKM factors bound to their target genes to an equal degree in both groups of cells. However, in non-iPSCs, the endogenous core stem cell genes were not activated, partially due to the lack of a promoter-enhancer intrachromosomal loop architecture 8, 9. We further demonstrate that the downstream enhancer and promoter of iPSC stemness genes are spatially connected through the formation of a chromatin inner loop structure 8, which is necessary for inducing the transformation to produce iPSCs 10, 11. In addition, maintaining pluripotency also requires the genome of pluripotent stem cells to be organized in the form of higher-order architecture 12-14. However, the molecular factors that orchestrate this pluripotency- specific intrachromosomal network are still poorly understood.

Long noncoding RNAs (lncRNAs) have recently been recognized as functional molecules taking part in epigenetic, transcriptional, and post-transcriptional control of gene expression 15-19. Some lncRNAs are differentially expressed and may control pluripotency and stemness, or they may promote differentiation of pluripotent cells 20-23. We have used an RNA reverse transcription-associated capture sequencing (RAT-seq) approach to characterize functional lncRNAs that interact with the *Oct4* promoter 24, a key transcription factor required for reprogramming somatic cells into iPSCs. The data from conventional RNA-seq were combined with RAT-seq to identify differentially-expressed lncRNAs that may be associated with intrachromosomal looping 25. Using this strategy, we identified a series of functional lncRNA candidates that are associated with pluripotency 24, 25. We characterized *Peblr20*, an *Oct4* enhancer binding lncRNA, as an essential chromatin factor for the maintenance of stem cell pluripotency. Notably, *Peblr20* controls stem cell pluripotency in *trans* by recruiting TET2 to the *Oct4* enhancer locus, thereby activating the enhancer for the initiation of reprogramming 26.

In the present study, we focused on the role of *Osblr8*, an *Oct4-Sox2* promoter-interacting lncRNA, in pluripotent reprogramming. We show that *Osblr8* is a pluripotency-associated lncRNA and is required for the maintenance of the stem cell pluripotent state. By interacting with multiple pluripotency-associated transcriptional factor genes, *Osblr8* epigenetically regulates their activity by coordinating pluripotency- specific intrachromosomal looping. This study highlights the role of *Osblr8* as a chromatin architecture modulator in the enhancement of reprogramming.

## Results

### CRIST-seq identifies *Osblr8* as an essential lncRNA for pluripotency

**Figure 1:**
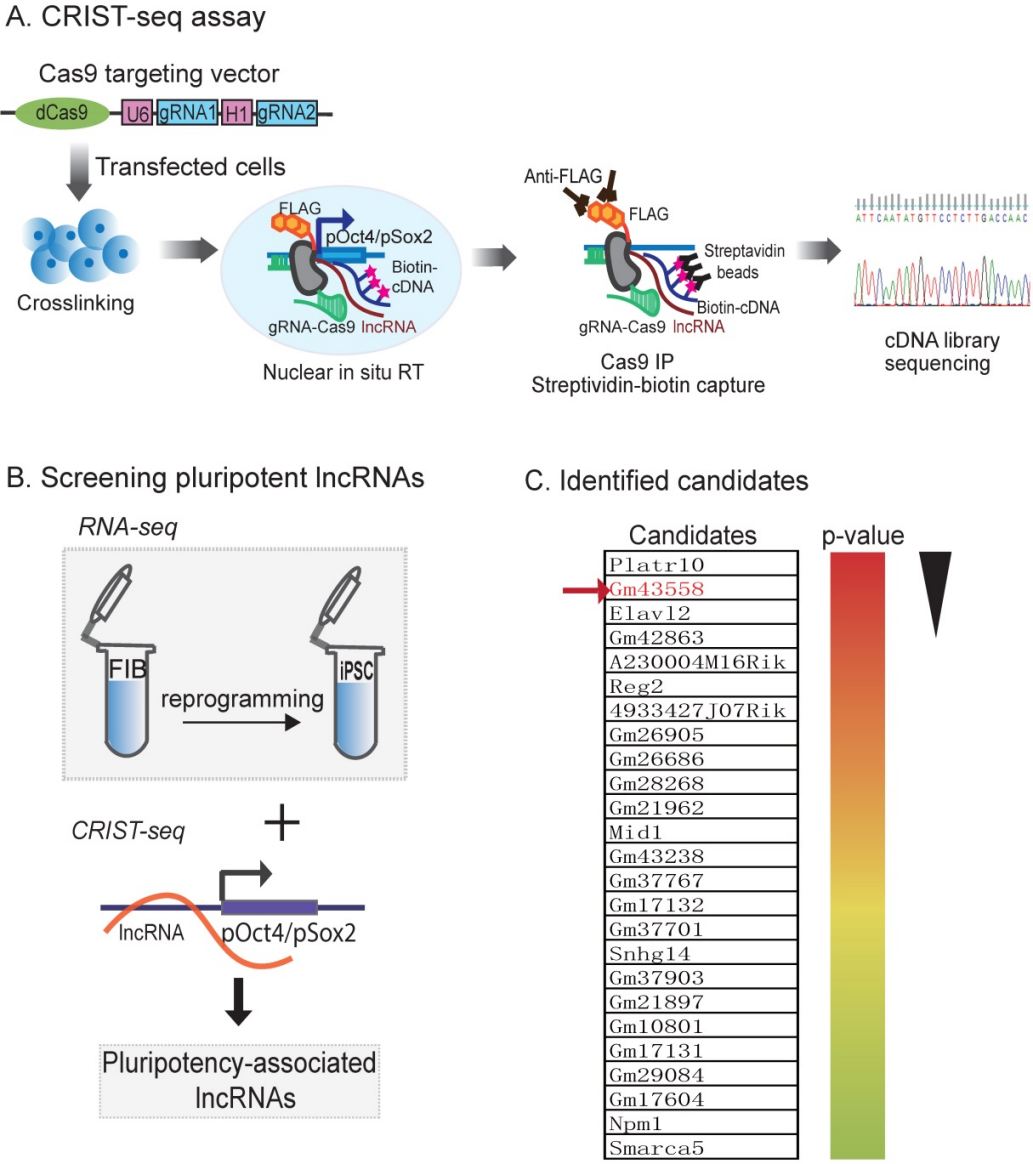
** Mapping pluripotency-associated lncRNAs by RNA-seq and CRIST-seq. A)** Chromatin-lncRNA *in situ* reverse transcription trap sequencing (CRIST-Seq) assay. dCas9: Catalytically inactive CRISPR Cas9; FLAG: a tag octapeptide at the N-terminal of Cas9; gRNA: Cas9 gRNAs that target the *Oct4/Sox2* promoter. After fixation, the *Oct4/Sox2* promoter-interacting RNAs were reverse transcribed into cDNAs in the isolated nuclei with biotin-dCTP. The Cas9 *Oct4*/*Sox2* promoter biotin-cDNA complex was immunoprecipitated by a Cas9-FLAG antibody, and biotin-cDNAs were further purified from genomic DNAs by biotin-streptavidin beads. The CRIST-captured cDNAs were used for Illumina library sequencing to identify the RNA components in the *Oct4* and *Sox2* promoters. **B)** Profiling pluripotency-associated lncRNAs by CRIST-seq and RNA-Seq. The *Oct4*/*Sox2*-interacting lncRNAs identified by CRIST-seq were integrated with RNA-seq data. The combination of these two datasets helps identify lncRNAs that interact with the *Oct4* and *Sox2* promoters and are also expressed differentially in reprogramming. **C)** Pluripotency-associated RNA candidates identified by RNA-Seq and CRIST-Seq. The RNA candidates are ranked on the basis of the RNA expression-fold between fibroblasts (FIBs) and iPSCs from the high (red) to the low (blue). Gm43558 (*Osblr8*) was chosen for further studies.

In order to explore the epigenetic mechanism in chromatin remodeling, we focused on the lncRNAs that interact with the promotor of core pluripotency maintenance factors *Oct4* or *Sox2*, two core pluripotency regulators in reprogramming. We proposed that lncRNAs embedded in or interacting with these loci were involved in the regulation of pluripotency. For this purpose, we used a CRIST-Seq approach 24 to profile lncRNAs that interact with the promoters of *Oct4* and *Sox2* **(Fig.1A).** This assay combines the simplicity of nuclear *in situ* RNA biotin labeling with the specificity of CRISPR Cas9 gene targeting. Cas9 gRNAs and control gRNA (gCT) were transfected into iPSCs, and chromatin-associated RNAs were *in situ* labelled by reverse transcription with biotin. After Cas9-FLAG immunoprecipitation and purification of the promoter-associated cDNAs from genomic DNAs by streptavidin beads, we constructed a cDNA library for Illumina sequencing.

We reasoned that an ideal pluripotent lncRNA candidate should also become activated in reprogramming. Therefore, we collected cells at different stages of reprogramming 27, 28, and RNA-Seq was performed to identify RNAs that were differentially expressed in association with reprogramming 25. To identify the pluripotency-associated lncRNAs candidates, we integrated the *Oct4* and *Sox2* CRIST lncRNA data with the RNA-Seq data **(Fig.1B)**. By combining these three datasets, we identified 25 RNA candidates that not only interacted with the *Oct4* and *Sox2* promoters but were also differentially activated during reprogramming **(Fig.1C)** 24.

Using this approach, we identified an *Oct4*/*Sox2* promoter-binding lncRNA ENSMUSG00000106628 as a pluripotency-associated lncRNA candidate. We referred it to as *Osblr8* (*Oct4*-*Sox2* binding long noncoding RNA 8) to better reflect its function in stem cells. *Osblr8* is a 210 bp long lncRNA located in chromosome 3 **(Fig.S1A)**; there was a large-fold increase in *Osblr8* abundance when fibroblasts were reprogrammed into iPSCs.

### LncRNA *Osblr8* is highly expressed in pluripotent stem cells

**Figure 2:**
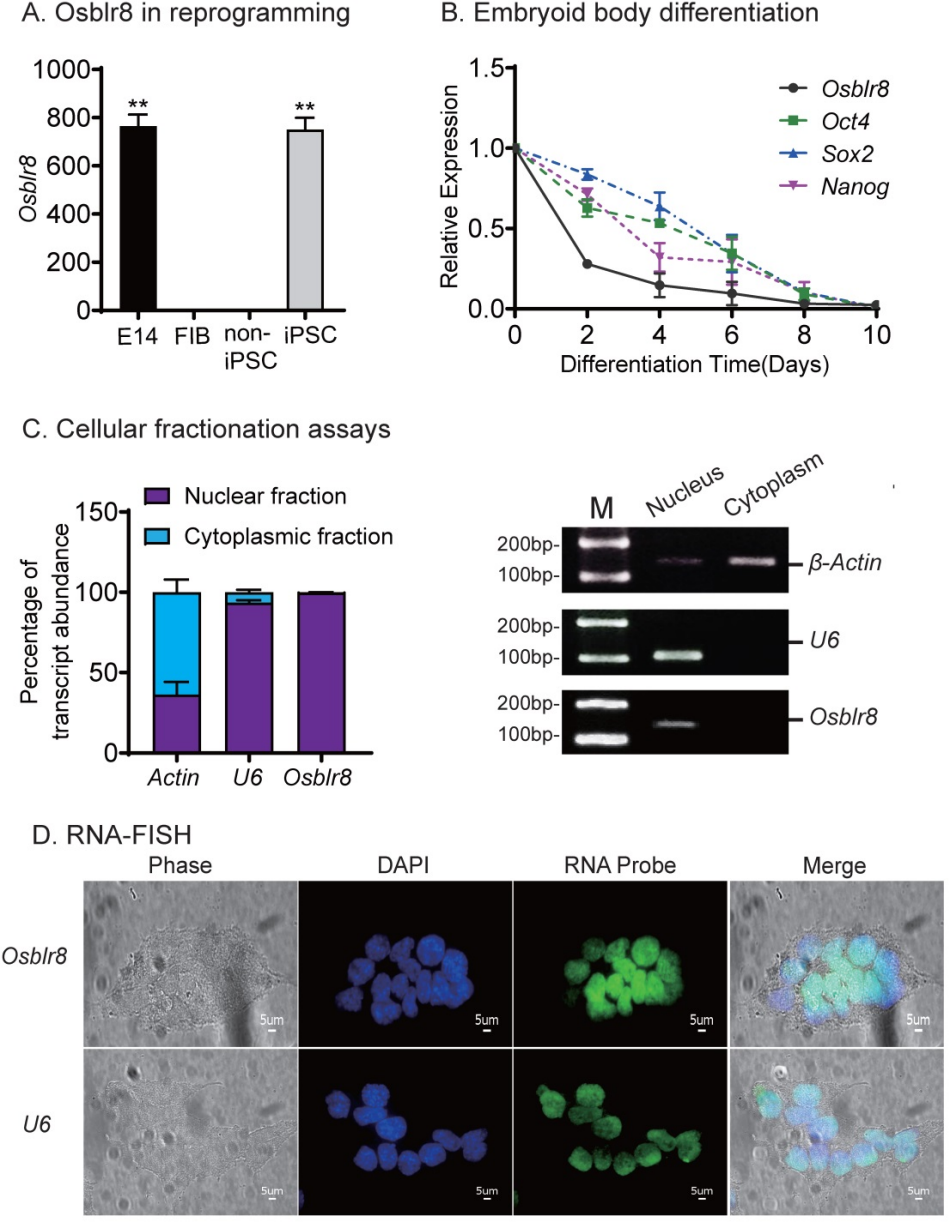
*** Osblr8* is a pluripotency-associated lncRNA. A)** Differential expression of* Osblr8* in reprogramming. Fib: fibroblasts; non-iPSC: unreprogrammed cells that express four OSKM cocktail factors but failed to complete reprogramming; iPSC: induced pluripotent stem cells; E14: mouse pluripotent stem cell line used as a positive control. Gene expression was measured by Q-PCR and normalized to β-Actin. For comparison, E14 embryonic pluripotent stem cells were used as the positive control. **B)** The expression of* Osblr8* is associated with *Oct4, Sox2, and Nanog* expression in embryoid body (EB) differentiation. iPSCs were collected at different stages of EB differentiation for quantitative PCR. **C)** Subcellular localization of* Osblr8* lncRNA. RNA extracted from each fraction was analyzed by Q-PCR. Data shown are mean ± SD (*n* = 3). β-Actin was used as the cytoplasmic control and U6 was used as the nuclear control. **D)** RNA FISH of* Osblr8*. LncRNA probes were synthesized using DIG-11-dUTP and detected by anti-digoxigenin-fluorescein (green). DAPI was used to stain the nucleus of iPSCs (blue).* Osblr8* lncRNA was predominantly located in the nucleus.

To determine the role of *Osblr8* in regulating pluripotency, we first verified its expression in different reprogramming stages, including fibroblasts, iPSCs, E14, and in non-iPSCs that expressed the lentiviral OSKM factors but failed to complete reprogramming. We confirmed that *Osblr8* was highly expressed in fully reprogrammed iPSCs and E14, whereas *Osblr8* was nearly undetectable in fibroblasts and non-iPSCs **(Fig.2A)**.

We also collected cells during the process of embryoid body differentiation and examined the expression of *Osblr8*. Using quantitative PCR, we found that *Osblr8* was significantly downregulated during embryoid body differentiation, showing a similar expression pattern with core stem cell factors *Oct4*, *Sox2*, and *Nanog* **(Fig.2B)**. Using cellular fractionation PCR, we found that *Osblr8* was predominantly located in the nucleus **(Fig.2C)**, and the nuclear localization was also confirmed by RNA FISH (**Fig.2D**).

### LncRNA *Osblr8* Is required for pluripotency maintenance of stem cells

**Figure 3:**
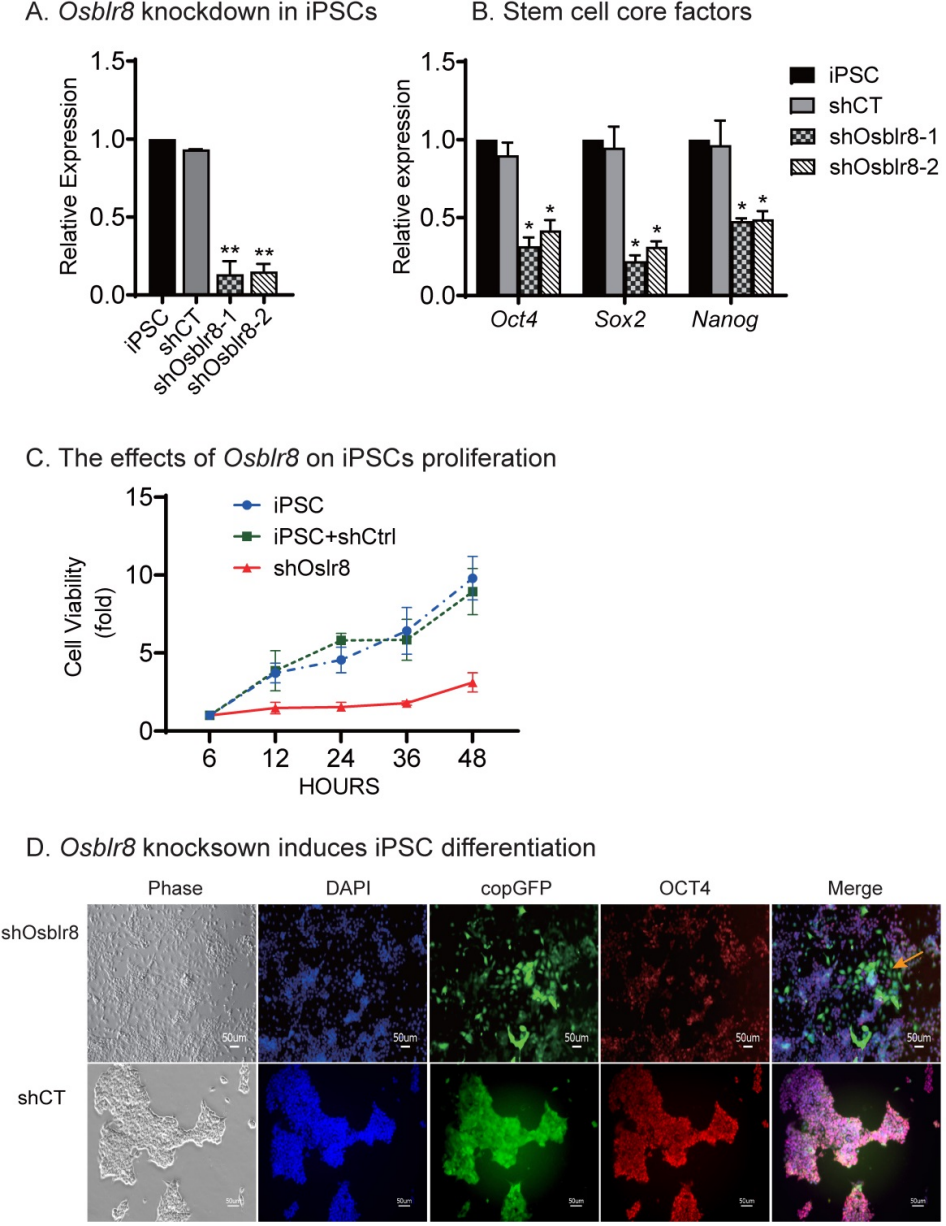
***Osblr8* is required for the maintenance of pluripotency. A)** *Osblr8* knockdown in iPSCs. After lentiviral shRNA transfection and puromycin selection, iPSCs colonies were selected and expanded for Q-PCR. shCT: random shRNA control; shOsblr8-1, -2: Lenti* Osblr8* shRNA-1 and* Osblr8* shRNA-2 vectors. For comparison, the abundance of* Osblr8* in iPSCs was set as 1. ** p<0.001 as compared with iPSCs and random shCT controls. **B)** *Osblr8* knockdown downregulates stem cell core factors in iPSCs. * p<0.05 as compared with iPSC and random shCT controls. **C)** Effects of* Osblr8* on iPSCs proliferation. MTT assay was performed to determine the viability of iPSCs transfected with shOsblr8, shCT and untreated iPSCs, respectively. **D)** *Osblr8* knockdown induces iPSC differentiation. The* Osblr8* shRNA and random shRNA lentiviral vectors carry the copGFP reporter gene (green). After lentiviral transfection, cells were fixed and immunostained by an antibody against the stem cell pluripotent marker OCT4 (red). Compared to the control group, shOsblr8 transfected iPSCs were negative for OCT4 immunostaining and were differentiated morphologically (yellow dotted line).

To characterize its role in pluripotency maintenance, we silenced *Osblr8* in iPSCs using two pairs of lentivirus-mediated short hairpin RNAs (shRNAs)**(Fig.S2A)**. After lentiviral transfection, iPSCs were selected by puromycin. Single colonies emitting the copGFP green signal were selected, expanded, and collected for Q-PCR. We found that both shRNAs achieved high knockdown efficiency **(Fig.3A)**. Notably, the depletion of *Osblr8* lncRNA significantly reduced the expression of pluripotency core factors *Oct4*, *Sox2*, and *Nanog* compared with scrambled control (shCT) cells and untreated iPSCs **(Fig.3B)**. Using an MTT assay, we examined the proliferation capacity of these cells and found that knockdown of *Osblr8* altered the proliferation capacity of iPSCs compared to iPSCs and the cells transfected with shCT **(Fig.3C)**.

We next examined if *Osblr8* knockdown would affect pluripotency of iPSCs **(Fig.3D)**. In the random shRNA control group (shCT), the copGFP-positive cells maintained the same cell morphology as pluripotent stem cells. However, knockdown of *Osblr8* dramatically altered cell morphology (top panel5, yellow arrow, shOsblr8). The *Osblr8*-knockdown cells became spindle-shaped and flat, appearing like fibroblasts. The pluripotency of treated iPSCs was examined by immunohistochemical staining of the pluripotency-associated marker protein OCT4. As expected, the shCT control group showed extensive expression of OCT4** (Fig.3D, bottom panel 4)**. After *Osblr8* shRNA knockdown, however, iPSCs became differentiated and lost the OCT4 expression (top panel 5, green area without red, yellow arrow). In the shOsblr8 group, there were some cells that escaped lentiviral infection and did not express the copGFP tack marker, and they maintained the original compact shape of iPSCs and expressed OCT4. Thus, lncRNA *Osblr8* knockdown causes stem cells to exit from the pluripotency state.

### LncRNA *Osblr8* enhances the activation of stem cell core factors

**Figure 4:**
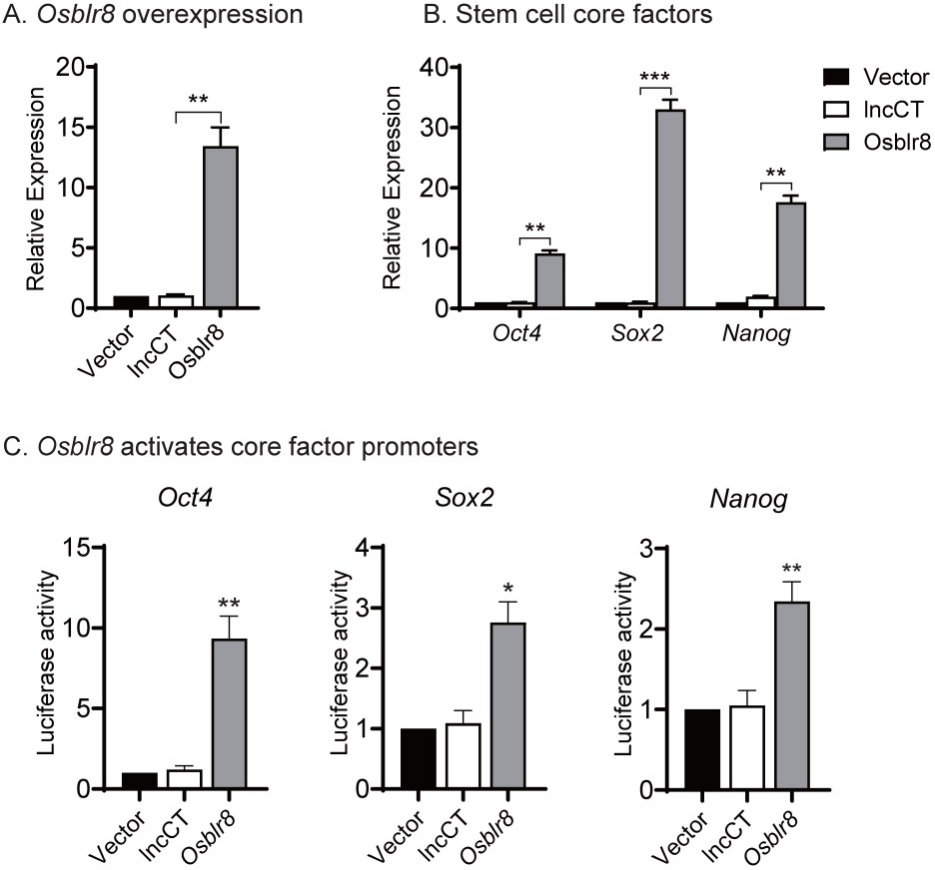
***Osblr8* activates stem cell core factor genes. A)** Lentiviral* Osblr8* overexpression in fibroblasts. Vector: empty lentiviral vector; lncCT: lncRNA random control;* Osblr8*: Lentiviral* Osblr8* overexpression. ** p<0.001 as compared with the Vector and lncRNA controls. **B)** Lentiviral overexpression of* Osblr8* activates the endogenous stem cell core factor genes. *** p<0.0001, ** p<0.001 as compared with the Vector and lncRNA controls. **C)** *Osblr8* activates promoter activities of core pluripotent factors. 293T cells were co-transfected by reporter plasmids and* Osblr8* plasmid. Forty-eight hours after transfection, cells were collected for luciferase activity measurements. Reporter plasmid empty vector and random lncRNA (lncCT) vectors were used as the controls. For comparison, the untreated fibroblasts were set as 1. * P < 0.05, and ** P < 0.001.

We then conducted a series of studies to explore the molecular mechanisms by which *Osblr8* regulates reprogramming. First, we examined if *Osblr8* was able to activate the expression of core stem cell factor genes in fibroblasts. We synthesized a lentipCMV-DsRed/ Puro-*Osblr8* plasmid **(Fig.S2B)** and packaged the lentivirus in 293T cells. Fibroblasts were transfected with *Osblr8* lentiviruses. After puromycin selection, ~60% of fibroblasts overexpressed *Osblr8* as assessed using DsRed as a tracking marker. Using Q-PCR, we confirmed a >10 fold overexpression of *Osblr8* as compared with the vector control **(Fig.4A)**. The endogenous *Oct4, Sox2* and* Nanog* genes were also upregulated in cells in which *Osblr8* was overexpressed **(Fig.4B)**.

Next, we explored whether *Osblr8* could activate the *Oct4*, *Sox2* and *Nanog* promoters. We synthesized three luciferase plasmids containing the promoters of these three master pluripotency transcript factors, respectively, which were then co-transfected with the *Osblr8* overexpression plasmid in 293T. Forty-eight hours after transfection, cells were harvested, and the luciferase activity was measured using the Dual-Luciferase reporter assay system. As shown in **Figure 4C**, the overexpression of *Osblr8* increased the luciferase activity of these promoters in 293T by 9, 2, and 2-fold greater, respectively.

### LncRNA *Osblr8* interacts with TET Family enzymes

We then focused on the epigenetic mechanisms by which *Osblr8* regulates *Oct4.* In order to initiate reprogramming, the methylated CpGs in the *Oct4* promoter must be demethylated to initiate transcription in somatic cells. The ten-eleven translocation (TET) family enzymes catalyze the stepwise oxidation of 5-methylcytosine in DNA to 5-hydroxymethylcytosine and play important biological functions in embryonic stem cells, development, aging, and disease 29. Thus, we examined if *Osblr8* regulates the *Oct4* gene through the TET family enzymes.

**Figure 5:**
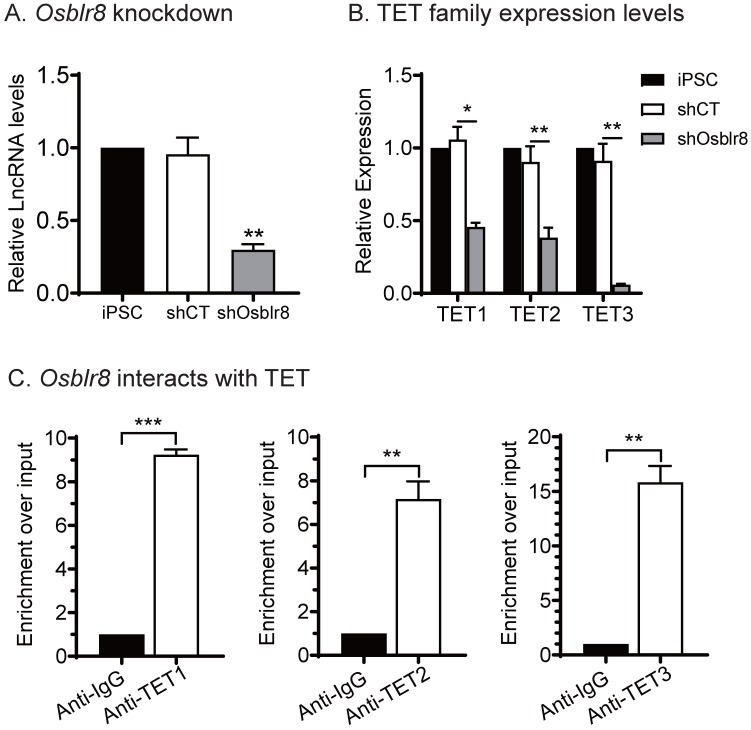
***Osblr8* interacts with TET protein family. A)** Knockdown of* Osblr8* in iPSCs. **B)** TET family expression in* Osblr8* knockdown iPSCs. cDNAs were extracted from iPSCs loss-of-function experiment. The TET1/2/3 primers were used for Q-PCR. **C)** Interaction of* Osblr8* with TET enzymes by RNA-chromatin immunoprecipitation (RIP). The TET-RNA chromatin complex was immunoprecipitated with an antibody against TET1, TET2 and TET3. After de-crosslinking, the immunoprecipitated RNAs were reverse transcribed. The TET-interacting* Osblr8* was measured by Q-PCR. IgG was use as the antibody control. Input: aliquot DNAs collected during the RIP assay. * P < 0.05, ** P < 0.01, and *** P < 0.001. NS: not significant.

We compared the expression of the TET family genes in iPSCs and *Osblr8*-knockdown iPSCs and showed that the expression of* Tet1, Tet2* and *Tet3* was downregulated in parallel with *Osblr8* knockdown **(Fig.5A and 5B)**, suggesting that *Osblr8* affects the TET family genes at the transcriptional level.

We then performed an RNA-binding protein immunoprecipitation (RIP) assay in iPSCs to examine if *Osblr8* interacts directly with TET1, TET2, and TET3 enzymes. The TET 1/2/3-RNA chromatin complex was immunoprecipitated by anti-TET1/2/3 antibodies, respectively. A mouse IgG was used as the negative control. The RNA pull-downs were reverse transcribed and detected by real-time Q-PCR using primers from *Osblr8*. As compared with the IgG control, we detected a marked enrichment of *Osblr8* in the TET antibody-precipitated complexes **(Fig.5C)**. These data suggest that *Osblr8* may activate the endogenous *Oct4* gene via TET-induced DNA demethylation.

### *Osblr8* orchestrates pluripotency-specific intrachromosomal looping

Chromatin remodeling is a major epigenetic barrier that determines the initiation of the pluripotency status in reprogramming 30-32. By comparing local chromatin structure of the *Oct4* locus, we previously revealed that there was a pluripotency-associated intrachromosomal loop in iPSCs that juxtaposes a downstream enhancer to the gene's promoter, enabling activation of endogenous stemness genes to achieve reprogramming 8. We asked whether *Osblr8* lncRNA participates in the orchestration of intrachromosomal looping for reprogramming.

**Figure 6:**
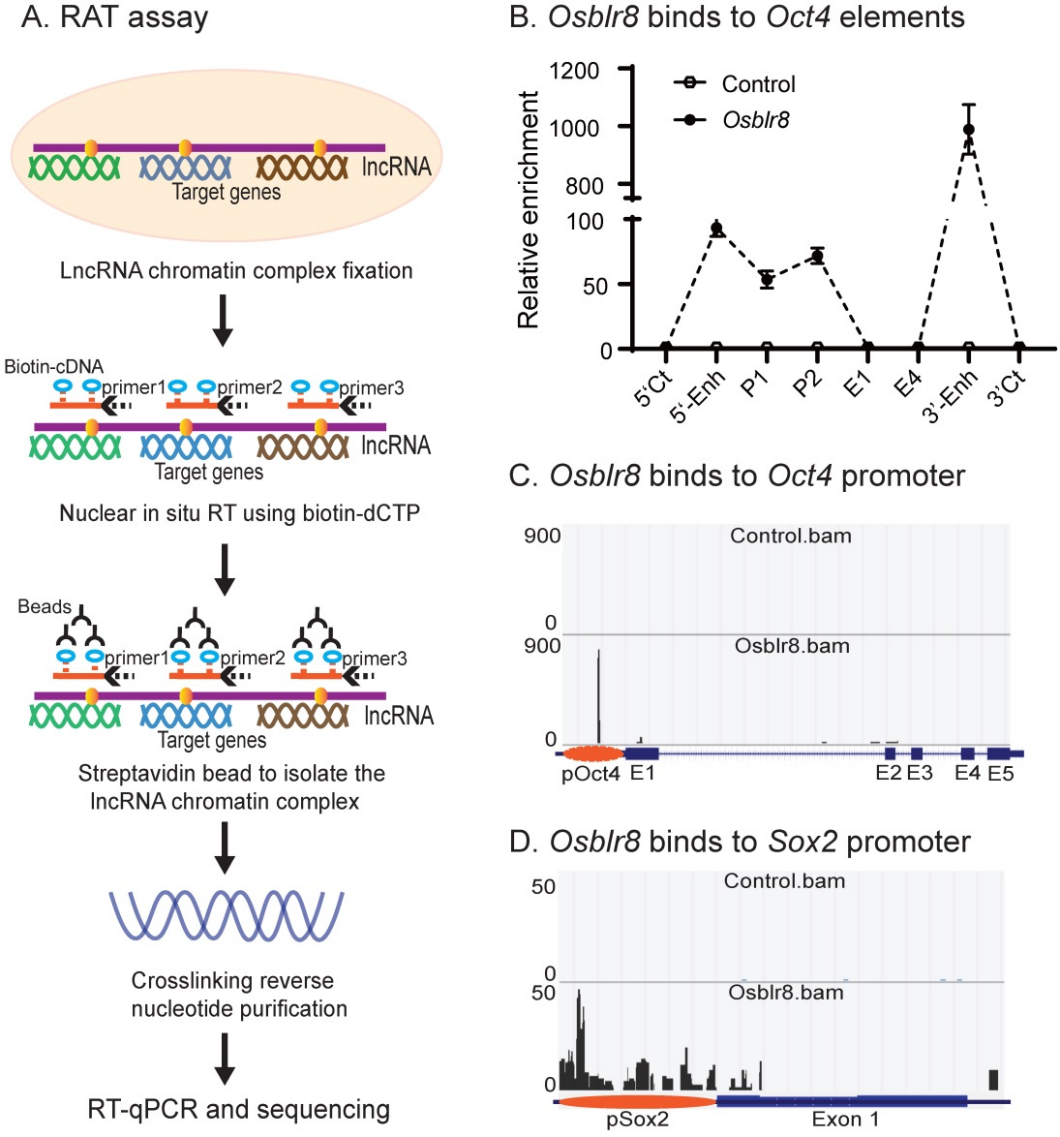
** Mapping the *Osblr8* interaction network by RAT-seq. A)** RNA reverse transcription-associated trap (RAT) assay. After fixation, *Osblr8* lncRNA was reverse transcribed into cDNAs in the isolated nuclei with biotin-dCTP using three gene specific reverse primers. The DNA-protein-lncRNA-biotin-cDNA complex was further purified from genomic DNAs by biotin-streptavidin beads. After the crosslinking reversal, the captured DNAs were used for Illumina library sequencing or analyzed by Q-PCR to identify the *Osblr8* interaction network. **B)** *Osblr8* lncRNA-*Oct4* DNA interactions by Q-PCR. RAT library samples were used to perform Q-PCR to quantitate binding intensity. The results were normalized to the value of the streptavidin bead pulldown control. Control: the RAT-seq library was constructed using random oligo primers; Obelr20: the RAT-seq library was constructed using *Osblr8*-speccific primers; 5'-CT, 3'-CT: the RAT 5'- and 3'- control sites; P: promoter; E1 and E4: Oc4 exons 1 and 5. **C)** Interaction of *Osblr8* at the *Oct4* locus. Control: the RAT library was constructed with random oligo primers. *Osblr8*: the RAT library was constructed using *Osblr8* complementary primers; 5'-Enh: 5'-enhancer; E1-E5: *Oct4* exons 1-5; 3'-Enh: 3'-enhancer. Note the enriched binding of *Osblr8* lncRNA at the *Oct4* promoter. **D)** Interaction of *Osblr8* at the *Sox2* locus. *Osblr8* binds to the *Sox2* promoter area.

We first examined the binding of *Osblr8* in the pluripotency-associated network. A detailed IGV analysis of the CRIST-seq data indicated that the signal of *Osblr8* binding to the *Oct4* and *Sox2* promoter was enriched only in the Cas9 *Oct4* and *Sox2* gRNA cells, but not in the Cas9 gCT control and the IgG immunoprecipitation control (**Fig.S3A**). The CRIST-seq IGV analysis revealed that *Osblr8* bound to the *Oct4* and *Sox2* promoter using two 50 bp fragments (5'-CCCCCTTCCTTCATAACTAGTGTCGCAACAATAAAATTTGAGCCTTGATC-3' and 5'-CCAGCCTGAAACCTGCTTGCTCGGGGTGGAGCTTCCTGCTCATTCGTTCT-3'). Using RAT-seq (**Fig. 6A**), we found that *Osblr8* bound to the *Oct4* promoter and to 5'- and 3'-enhancer elements (Fig. 6B and 6C). The RAT-seq IGV analysis also showed that *Osblr8* binds to the *Sox2* promoter. No such interaction signals were detected in the RAT random control library products (CTL).

**Figure 7:**
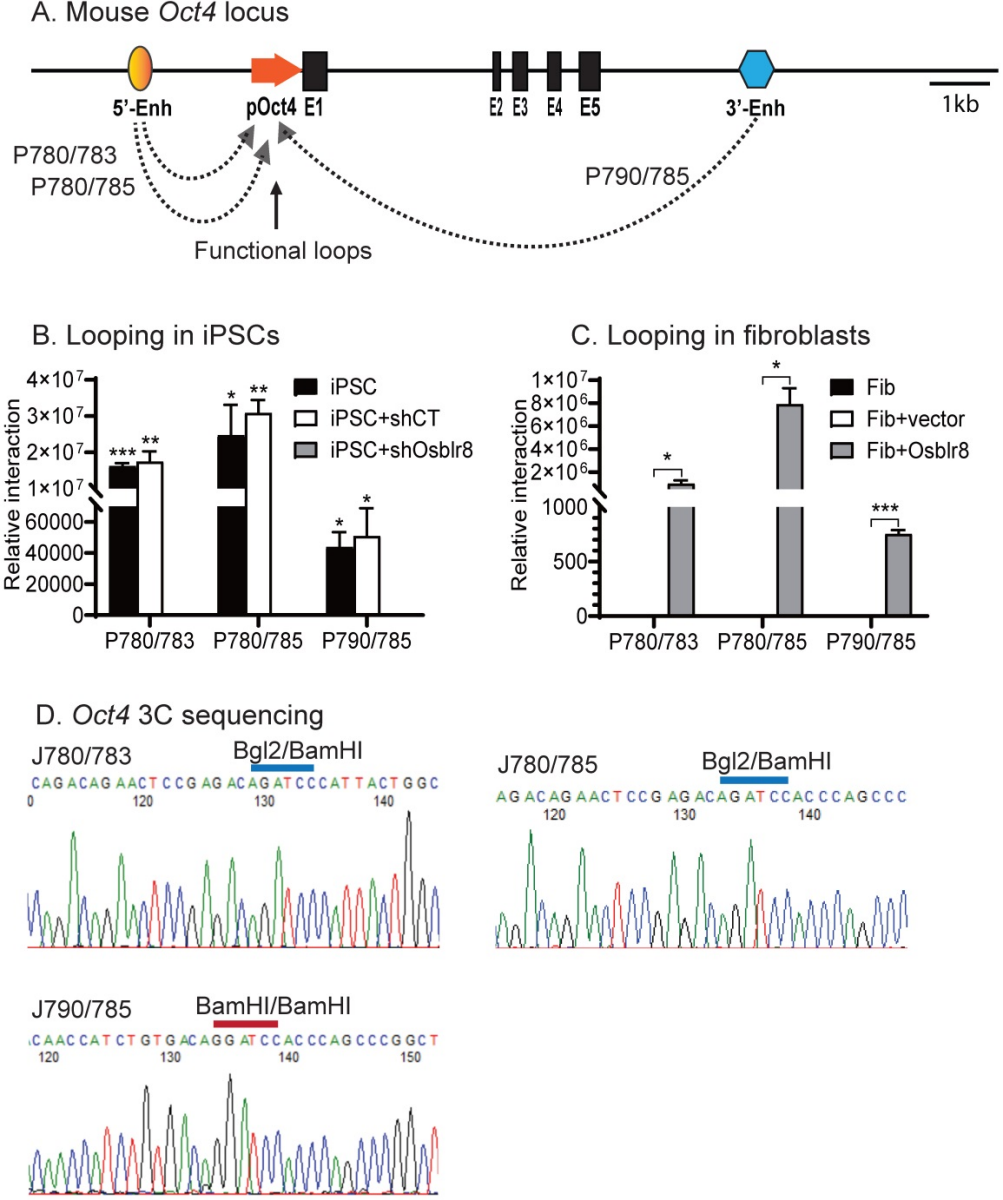
*** Osblr8* orchestrates pluripotency-specific intrachromosomal looping. A)** Location of 3C primers used to detect the interaction between the *Oct4* promoter and enhancer. Enh: enhancers; p*Oct4*: *Oct4* promoter; E1-E5: exons; Arrows: intrachromosomal interactions. **B)** Knockdown of *Osblr8* abolishes the intrachromosomal interaction loop. shCT: negative control shRNA, shOsblr8: shRNA that targets* Osblr8* lncRNA. iPSC: induced pluripotent stem cells. Primer sets that detect the presence of looping are marked in Fig.7A. The 3C interaction was quantitated by qPCR and was standardized over the 3C control Ercc3 gene. For comparison, the relative 3C interaction was calculated by setting the 5' or 3' control as 1. *P<0.05, ** P< 0.01 as compared with the shOsblr8 treatment and shRNA control. **C)** Overexpression of* Osblr8* induces *de novo* formation of intrachromosomal looping. FIB: fibroblasts. Fib+Vector: fibroblasts transfected with vector control; Fib+*Osblr8*: fibroblasts overexpressed* Osblr8.* **D)** Sequencing of the *Oct4* intrachromosomal loop products. Blue line on the top of the sequence: the 3C ligation product between the BamH1 and Bgl2 sites. Red line on the top of the sequence: the 3C ligation product between the BamH1 and BamH1 sites.

We then used chromatin conformation capture (3C) 33, 34 to compare the intrachromosomal looping between iPSCs, shRNA control cells, and *Osblr8* knockdown cells. Cells were fixed with 1% formaldehyde, digested with restriction enzymes BamH1/BglII, and then connected with T4 DNA ligase. After crosslinking reversal and DNA purification, chromatin interactions were detected by specific 3C primers located in the promoter and the enhancers of *Oct4* (**Fig.7A**). As previously reported 8, we detected reprogramming-associated intrachromosomal interaction products in iPSCs: the P780/ P783 and P780/P785 loops between the 5'-upstream enhancer and the promoter, and the P790/P785 loop between the 3'-enhancer and the promoter **(Fig.7B)**. In the control iPSCs transfected with shRNA control (shCT), these intrachromosomal loops were intact. However, shRNA knockdown of *Osblr8* abolished these intrachromosomal interaction signals and caused the iPSCs to exit from pluripotency.

We also observed *de novo* formation of intrachromosomal interactions in fibroblasts when* Osblr8* was overexpressed in these cells **(Fig. 7C)**. We sequenced the 3C products and verified the presence of the ligated BamHI or BamHI/BglII sites, which were flanked by the sequences from the promoter and enhancers of *Oct4*, respectively **(Fig.7D)**. These data suggest that *Osblr8* is critical in the maintenance of intrachromosomal interactions that are known to associate with reprogramming and maintenance of pluripotency 8.

## Discussion

Formation of a specific promoter-enhancer intrachromosomal architecture constitutes a critical epigenetic barrier in pluripotent reprogramming 8, 35. In this study, we focused on the chromatin factors that interact with the *Oct4* and *Sox2* promoters. Using CRIST-seq, we identified *Osblr8* as a crucial regulator that helps maintain the pluripotency of iPSCs. *Osblr8* was differentially expressed during the reprogramming progress, silenced in fibroblasts, but highly expressed in iPSCs and E14 cells. The loss of *Osblr8* results in the exit of E14 cells from pluripotency, while an increased abundance of *Osblr8* activates the stem cell core factors in fibroblasts. Most importantly, *Osblr8* participates in the formation of a loop structure that is required for the activation of stemness gene expression. Loss of *Osblr8* abolishes the long-range interaction and leads to cell differentiation. Thus, *Osblr8* plays a crucial role in coordinating a topological architecture network that is necessary for maintenance of pluripotency.

Subcellular localization predicts the function of lncRNAs 36, 37. Nuclear lncRNAs usually act as guiders and tethers to enhance or activate the expression of specific genes. They can help chromatin modification complexes bind to specific genomic loci or act as scaffolds to tether together distinct related functional complexes 38, 39. Nuclear lncRNAs can interact with proteins and RNAs by base-pairing with other nucleic acids to activate or repress the transcription of defined genes 39-41. LncRNAs localized in the cytoplasm, on the other hand, usually regulate gene expression by base-pairing complementary regions on target RNAs 42. *Osblr8* is highly expressed in the nucleus and functions by binding to the *Oct4* promoter and interacting with TET proteins. *Osblr8* coordinates the formation of intrachromosomal loops, thereby enhancing the expression of stemness-related genes. Through this complex network, *Osblr8* can control the pluripotent state of iPSCs.

DNA methylation plays an important role during reprogramming. Inhibition of DNA methyltransferase DNMT1 can enhance the efficiency of reprogramming 43. The CpG methylation status of the promoter is critical in the regulation of the *Oct4* gene, as demonstrated by the different expression patterns seen in somatic and pluripotent cells 44-46. TET proteins can successfully induce DNA demethylation by the oxidation of C-5 position of cytosine (5mC) to 5-hydroxymethylcytosine (5hmC), 5-formylcytosine (5fC), and 5-carboxylcytosine (5caC). 5fC and 5caC are selectively recognized and excised by thymine DNA glycosylase (TDG), leading to DNA demethylation 47, 48 These pathways could allow turnover or resetting of DNA methylation to promote and maintain pluripotent states. TET1 and TET2 are highly expressed in mESCs, which is consistent with the relatively high levels of 5hmC and detectable levels of 5fC and 5caC in these cells 49-51. In the absence of TET1 and TET2, ESCs are intrinsically blocked from reaching complete global hypomethylation 52. Numerous studies show that the TET protein family plays an important role in maintaining pluripotency status. Through the CRIST-seq and RIP-PCR, we demonstrate that *Osblr8* may help recruit the TET protein family to the *Oct4* promoter, where DNA demethylation promotes the expression of the *Oct4* gene.

The 3D chromatin architecture is very important for transcriptional regulation. Looping between core promoter elements and distal enhancer or insulator elements controls the transcriptional activation or repression of genes, respectively 53-55. Some studies reported that Mediator and Cohesin physically and functionally connect the enhancers and core promoters of active genes in embryonic stem cells. Mediator, a transcriptional coactivator, forms a complex with cohesin, which can form rings that connect two DNA segments 56, 57. At the *Oct4* gene locus, for example, the intrachromosomal loops between the gene promoter and enhancer regulatory regions are specific for pluripotent stem cells. This topological structure helps bring distal regulatory elements, like enhancers, into physical proximity with gene target promoters, thereby activating them to initiate cellular reprogramming 8. In this study, we showed that *Osblr8* was enriched in the promoter and 5'- and 3'-enhancers of *Oct4*. A similar mechanism has been observed for a well-known lncRNA *Xist*, which initiates X-chromosome inactivation (XCI) in females by coating the inactive X-chromosome in *cis* 58-62. We demonstrated that *Osblr8* was essential for the maintenance of this intrachromosomal looping. Knockdown of *Osblr8* abolishes this intrachromosomal loop structure, and causes the exit of iPSCs from pluripotency. Thus, during reprogramming, *Osblr8* is actively transcribed and acts in concert with other chromatin factors to coordinate a topological architecture network that is necessary to initiate pluripotency. In addition to *Oct4*, our RAT-seq data show that *Osblr8* also binds to other pluripotency- associated factor genes, like *Sox2*. It will be interesting to explore if *Osblr8* also utilizes a similar mechanism to regulate the expression of *Sox2* in reprogramming.

Overall, our study identifies* Osblr8* as a novel pluripotency-associated lncRNA.* Osblr8* becomes activated in reprogramming. Depletion of *Osblr8* caused E14 cells to differentiate and lose pluripotency. *Osblr8* maintains stem cell pluripotency through multiple epigenetic mechanisms, including activation of endogenous stem cell core factor genes and coordination of intrachromosomal looping. *Osblr8* also regulates TET protein family genes, with which to control the expression of stemness genes. Thus, lncRNA *Osblr8* exerts its function as a chromatin epigenetic modulator in the regulatory network of stem cell pluripotency and reprogramming.

## Material and Methods

### CRIST-Seq to map the *Oct4*/*Sox2*-interacting lncRNAs

The CRIST-seq method used to identify lncRNAs that bind to the *Oct4* and *Sox2* promoters was described by Zhang et al 24. After CRIST-seq, the called peaks that overlapped with the IgG control enriched regions were removed, and the CRIST-Seq signal intensities were further normalized over that of the non-targeting Cas9 gCT control using parameters of fold-change difference ≥2 and p-value < 0.05, with false discovery rate (FDR) <0.1. The adjusted CRIST-Seq data were then used for mapping the *Oct4* and *Sox2* RNA interactions 24, 25.

### Identification of lncRNAs by RNA-seq in reprogramming

Mouse fibroblasts were reprogrammed with *Oct4*-*Sox2*-Klf4-c-Myc (OSKM) lentiviruses 8, 63. The isolated iPSC colonies were characterized by immunostaining stem cell markers, alkaline phosphatase staining, karyotype analysis, and teratoma formation. The fibroblast-like cells that expressed OSKM but were not reprogrammed were termed “non-iPSCs” and used in parallel with iPSCs in the study 8, 25. The lncRNAs that are differentially expressed in reprogramming were identified by RNA-seq. RNAs that are differentially expressed in reprogramming were identified using the fold-change > 2 and p < 0.05 with an unpaired two-sided t-test 25.

### RNA extraction and cDNA synthesis

Total RNA was extracted by Trizol reagent (Invitrogen). The concentration and quality of all RNA samples were evaluated by Nanodrop 1000 (ThermoScientific, CA), and the 260/280 and 260/230 values of all samples were more than 1.8 and 1.9, respectively. The extracted RNA samples were stored at -80°C, and cDNAs were synthesized using M-MLV reverse transcriptase (Invitrogen). Briefly, 400 - 800 ng total RNA was added to 12 µl liquid wax per reaction; we then used DNase I (Millipore Sigma, MA) to remove genomic DNA contamination. The reverse transcription reaction was performed with M-MLV reverse transcriptase at 37°C for 1 h, followed by 95°C for 10 min. After 10-fold dilution, cDNA was stored at -20°C or used for PCR and RT-qPCR.

### Quantitation of gene expression by Q-PCR

Real-time PCR was carried out using 3 X Klen-Taq I Mix with a Bio-Rad Thermol Cycler. PCR amplification was performed by PCR of 1 cycle at 98°C for 5 min, 32 cycles at 95°C for 20 s, 62°C for 15s and 72°C for 15 s, and 1 cycle at 72°C for 10 min. β-Actin was used as PCR input. Quantitative real-time PCR (Q-PCR) was performed using the FastStart Universal SYBR Green Master mix (Millipore Sigma, MA) with a StepOnePlus real-time PCR system (ABI Prism 7900HT; Applied Biosystems, USA). For quantitative real-time PCR, the threshold cycle (Ct) values of target genes were normalized over the Ct of the β-Actin control. Primers used for real-time PCR and qPCR are listed in **Table S1**.

### Preparation of Cytoplasmic and Nuclear Fractions

Cells were briefly digested by Trypsin-EDTA and gently resuspended in DMEM. After completely aspirating the PBS, 800 μl hypotonic buffer (10 mM Hepes, pH 7.9, 1.5 mM MgCl_2_, 10 mM KCl) were added and placed on ice for 2 min. 10% Nonidet P-40 was added to a final concentration of 0.4% (35 μl). Samples were inverted a few times and spun at 3,000 g for 7 min. Supernatants (cytoplasmic fractions) were collected for processing, and the pellet (nuclear fraction) was gently resuspended in 500 μl hypotonic buffer and spun at 3,000 g for 2 min. This washing step was repeated three to four times. Both the cytoplasmic and nuclear fractions were processed for RNA extraction, cDNA synthesis and real-time PCR or qPCR. To verify that the cytoplasmic and nuclear fractions were completely separated, we used U6 as a nuclear control and β-Actin as cytoplasmic control. The primers for PCR are listed in **Table S1**.

### RNA FISH

RNA FISH was performed by a modification of the method published previously 64-66. The RNA FISH probe was prepared as an antisense single strand DNA (ssDNA) by asymmetric PCR 67. Briefly, the ssDNA probe was synthesized by 3x Klen-Taq I DNA polymerase mix, the iPSC cDNA was used as the template, PCR primers were JH6195 and JH6196 as listed in **Table S1**. The DNA probe was purified by electrophoresis on 2% agarose gel and eluted in 20 μl TE buffer. For hybridization, 0.1 μg ssDNA probe and 10 μg salmon sperm DNA (Boehringer, Meylan, France) were precipitated with ethanol and suspended in 10 μl RNA hybridization buffer (2xSSC, 10% dextran sulfate, 0.2mg/mL BSA (Invitrogen, CA), 2mM VCR, 10% formamide). After sequential RNA FISH, slides were counterstained with DAPI, and FISH signals were detected using the Keyence BZ-X710 fluorescence microscope with GFP filter (EX 470/40 nm, DM 495nm, BA 525/50nm), Cy5 filter (EX 620/60 nm, DM 660nm, BA 700/75nm) and DAPI filter (EX 360/40 nm, DM 400nm, BA 460/50nm), respectively. Images were captured and merged to confirm the subcellular localization.

### Lentiviral overexpression of *Osblr8* lncRNA in fibroblasts

Full-length* Osblr8* lncRNA was amplified with PCR primers containing the EcoRI and EcoRV restriction sites. The PCR products were gel-purified, cut by restriction enzymes, and ligated into the pCMV-DsRed/Puro vector constructed in our lab. The* Osblr8* lncRNA clone was confirmed by sequencing and then packaged in 293T packing cells 68 using the method described in our lab 48. After transfecting fibroblasts, cells were selected by puromycin. The DsRed reporter in the vector was used to track lentivirus transfection efficiency. After 14 days, cells were collected for next experiments.

### MTT assay

Cell proliferation was quantitated using the Cell Growth determination KIT MTT Bases (Sigma, USA, Stock No. CGD-1) following the manufacturer's instructions. Cells are cultured in 96-well plates. When cells were adherent to the plate, medium was removed and MTT SOLUTION was added in an amount equal to 10% of the culture. Cells were incubated in a 37°C incubator with 5% CO_2_ for 4 h. Then the MTT SOLUTION was removed and the MTT SOLVENT was added in an amount equal to the original culture volume. After gentle shaking, the absorbance was spectrophotometrically measured at a wave length of 570 nm. We measured cell number at 6, 12, 24, 36, and 48 h. Cells were cultured in three wells and the study was repeated for three replicates.

### Knockdown of *Osblr8* lncRNA in iPSCs

LncRNA* Osblr8* was knocked down by two shRNA lentiviruses. The shRNA vector was constructed by cloning two shRNAs into pGreenPuro vector (#SI505A-1, SBI, CA). shRNAs were designed online (http://katahdin.cshl.edu/homepage/siRNA/RNAi). For cloning, two pairs of shRNAs (5'- CTGGAACCTGAGGAGCCACACACGT-3' and 5'- TGCACCTTTCTACTGGACCAGAGAT-3') combined with loop were linked to the H1 and U6 promoter using PCR and were ligated into the EcoR1/BamH1 site in pGreenPuro vector. The copGFP reporter in the vector was used to track lentivirus transfection in iPSCs. A random shRNA (GCAGCAACTGGACACGTGATCTTAA) was cloned in the same vector as the assay control (shCT). After lentiviral transfection, iPSCs were selected by puromycin. Single colonies were selected and cultured for expansion. Cells were collected for RNA quantitation of* Osblr8* lncRNA and related genes using RT-qPCR.

### Immunofluorescent staining of stem cell markers

Immunofluorescent staining was used to examine stem cell markers in iPSC colonies 69. Briefly, cells were fixed by freshly made 4% paraformaldehyde for 10 min at room temperature, permeabilized with freshly made 0.5% v/v Triton X-100/PBS on ice for 5min, then blocked in 1% w/v BSA for 30 min at room temperature. After incubation with primary antibodies diluted in 1% BSA (2 μg/ml 1:500) for 1-3 h at room temperature, samples were washed three times in PBS for 5 min each, and then incubated with the secondary antibodies for 1 h at room temperature. The following antibodies were used in the immunostaining: rabbit anti-OCT4 (1:100 dilution, Santa Cruz). The cell samples were subsequently incubated with Cy3 or Alexa Fluor 647 labeled secondary antibodies for 1 h. After washing three times with PBS, samples were counterstained with Hoechst 33258 (Invitrogen). Alternatively, the pluripotency of stem cells was examined by Fluorescent Mouse ES/iPS Cell Characterization kit (Cat.#SCR077, Millipore, MA) following the protocol provided by the manufacturer. Fluorescence images were acquired with a Zeiss AxioCam Camera.

### Embryoid body differentiation

E14 cells were cultured by the hanging drop method in a 10-cm culture dish without LIF. After 3 days, EBs were transferred to 10-cm ultra-low-adherence plates for suspension culture with slow shaking for up to 12 days. The media were replenished by sedimentation every other day. Embryoid bodies were collected on D0, D2, D4, D6, D8, D10 and D12 for quantitation of* Osblr8* lncRNA and targeted genes using RT-qPCR.

### Luciferase assay

The function of* Osblr8* in activating the promoters was first examined in 293T cells by using a dual-luciferase reporter assay. A 3.9 kb genomic DNA fragment covering the *Oct4*,* Sox2 and Nanog* promoters respectively. The promoter DNA fragment was cloned into pGL3 vector by Kpn1/Xho1.

For the luciferase assay, cells were seeded at a density of 5 × 10^4^ cells/well in 24-well plates. The lentiviral* Osblr8* overexpression vector was co-transfected with an *Oct4*-luciferase plasmid and Renilla luciferase control plasmid (Promega) using Lipofectamine 3000 (Invitrogen, CA). The empty lentiviral vector and random lncRNA vector were used as controls. Forty-eight hours after transfection, firefly and Renilla luciferase activities were measured with the dual-luciferase reporter system (Promega) using a luminometer (Turner Biosytem, CA). The relative activity of the promoter was calculated by setting the untreated control cells as 1. All luciferase assays were repeated three times with three culture replicates each.

### Profiling the *Osblr8* genome-wide gene targets by RAT-seq

The RAT-seq approach was used to map the genome wide interacting target genes for lncRNA candidates^25^, ^70^, ^71^. Briefly, 1.0 × 107 cells were cross-linked with 2% formaldehyde and lysed with cell lysis buffer (10 mM Tris [pH 8.0], 10 mM NaCl, 0.2% NP-40, 1X protease inhibitors). Nuclei were collected, suspended in 1X reverse transcription buffer in the presence of gene-specific primer, biotin-14-dCTP, RNase inhibitor and Maxima Reverse Transcriptase (Thermo Fisher Scientific, CA). After 30 min of reverse transcription of *Osblr8* lncRNA labeled by biotin-14-dCTP with Maxima Reverse Transcriptase at 65◦C. After nuclear lysis, the complex was sonicated for 180s (10s on and 10s off) on ice by 2-mm microtip at 40% output control and 90% duty cycle settings. The biotin-cDNA/chromatin DNA complex was pulled down with biotin-streptavidin magic beads (Invitrogen, CA). After reversing the cross-links and washing with 10 mg/ml proteinase K at 65°C overnight and treatment with 0.4 μg/ml RNase A for 30 min at 37°C, the genomic DNA that interacts with the lncRNA was extracted and digested by MboI, and ligated with the NEBNext adaptors (NEBNext® ChIP-Seq Library Prep Master Mix Set for Illumina) to construct the library. The library DNAs were used to Illumina sequencing (Shanghai Biotechnology, Shanghai) and binding PCR with primers shown in **Table S1**. We performed a RAT assay with random primers and constructed a control library for sequencing using the same protocol.

After RAT sequencing, the low quality reads were filtered using Fastx (version:0.0.13) software (http://hannonlab.cshl.edu/fastx_toolkit/index.html). Clean reads were mapped to the mouse genome (genome version: mm10) using the Bowtie (version:0.12.8) software with default parameters^72^. Enriched regions of the genome were identified by comparing the RAT-Seq peaks to input samples using MACS2 (version:2.1.1) and q-value of 0.05 was used as the initial cutoff threshold to minimize peak caller bias^73^. The upstream 2 k of the transcription start sites and the downstream 5k of the transcription termination region were defined as the gene regions. The significant GO terms of biological processes with a p-value < 0.05 were selected. We also used the MEME suite 74 for the discovery and analysis of the peaks' sequence motifs. The resulting coverage tracks (bedgraph file) were visualized in the UCSC genome browser. To reduce the background, the RAT-Seq data were further normalized over the peaks of the control RAT-Seq data that were generated by using random oligonucleotide primers in the RAT assay. Differential binding analysis was performed with the DiffBind package using parameters of fold change difference ≥2 and p-value < 0.05, with false discovery rate (FDR) <0.1. The adjusted RAT-Seq data were used for mapping the lncRNA target gene interaction network.

### Chromosome conformation capture (3C)

We used a 3C assay to determine intrachromosomal interactions 75, 76. Briefly, fibroblasts and iPSCs were cross-linked with 2% formaldehyde and lysed with cell lysis buffer. An aliquot of nuclei (2×10^6^) was digested with 800 U *BamH*I/*Bgl*II at 37°C overnight. Chromatin DNA was diluted with NEB ligation buffer and ligated with 4,000 U of T4 DNA ligase. After reversing the crosslinks, DNA was purified and used for PCR amplification using primers that are derived from different regions of the *Oct4* locus. The 3C PCR products were sequenced to validate the intrachromosomal interaction by affirming the presence of the *BamH*I/*Bgl*II or *BamH*I ligation site.

### RNA binding protein immunoprecipitation (RIP) assay

A lncRNA-affinity binding precipitation assay (RIP) 77 was performed to examine the binding of TET1/2/3 protein with* Osblr8* lncRNA. RIP was performed using the Magna RIP^TM^RNA-Binding Protein Immunoprecipitation Kit (Millipore, Germany) according to the manufacturer's instructions.* Osblr8*-overexpressed fibroblasts were collected and lysed using RIP lysis buffer. Then 100 μl cell extract was incubated with RIP buffer containing magnetic beads conjugated with anti-TET1/2/3 antibody (Abcam, MA). Mouse IgG was used as the negative control. The samples were incubated with proteinase K to digest protein, and then the immunoprecipitated RNA was isolated. The purified RNAs were sequenced and detected by reverse transcription qPCR. The primers for Q-PCR are listed in Supplementary **Table S1**. CHIP-qPCR was performed with three replicates. The Ct values were normalized over the input and compared with the IgG control.

### Statistical analysis

All experiments were performed in triplicate. The data were expressed as mean ± standard error of mean (SEM) and were analyzed using SPSS software (version16.0, IL). The data were analyzed with Student's *t*-test or by one-way analysis of variance, and statistically significant differences by Student's t test.

### Data availability

The RNA-Seq data generated in this study have been deposited in NIH GEO databases with accession number GSE116605, including 1). PSC RNA-seq.fq.gz (GSM3243668, iPSC RNA sequencing fastq data); 2). FIB RNA-seq.fq.gz (GSM3243669, Fibroblast RNA sequencing fastqdata) 25. The CRIST-Seq data generated in this study have been deposited in NIH GEO databaseswith accession number GSE107945. The folder contains four raw data files, including1). Cas9 *Sox2*-gRNA.fq.gz (*Sox2* promoter CRIST lncRNA sequencing fastq data); 2).Cas9 *Oct4*-gRNA.fq.gz (*Oct4* promoter CRIST lncRNA sequencing fastq data); 3).Cas9-gCT2.fq.gz (*Oct4* promoter CRIST Cas9-gCT random control library sequencingfastq data); 4). Cas9-IgG.fq.gz (CRIST IgG control library sequencing fastq data) 24.

## Supplementary Material

Supplementary figures and table.
