# Supplementary material for: Osblr8 orchestrates intrachromosomal loop structure required for maintaining stem cell pluripotency
Source: Int J Biol Sci. 2020 Apr 6;16(11):1861–75. doi: 10.7150/ijbs.45112 (PMC7211171; doi:10.7150/ijbs.45112)
Supplement: Supplementary file 1 — Supplementary figures and table. [file ijbsv16p1861s1.pdf]

A. *Osblr8* expression by RNA-seq

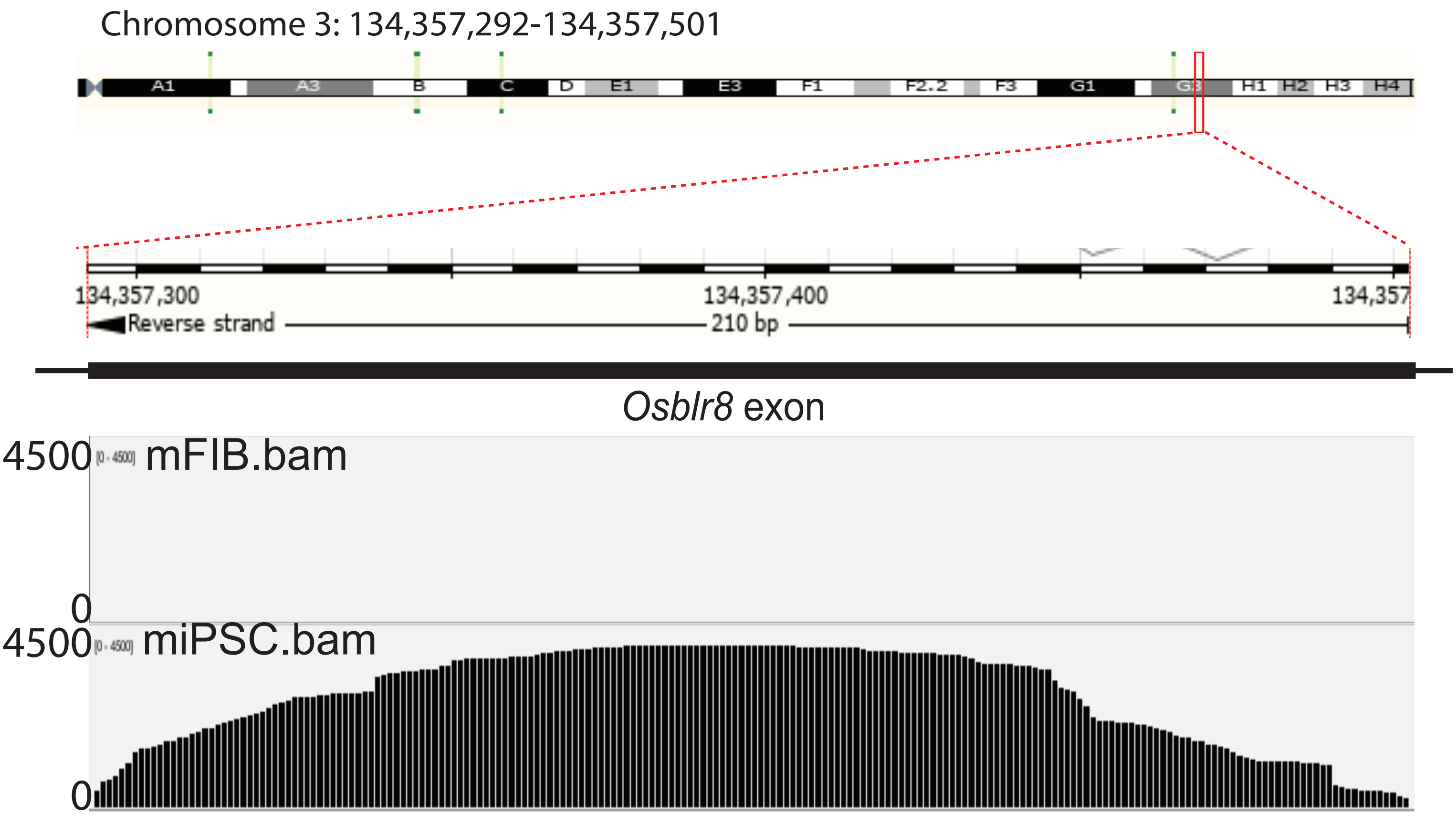

Figure S1. *Osblr8* is highly expressed in pluripotent stem cells

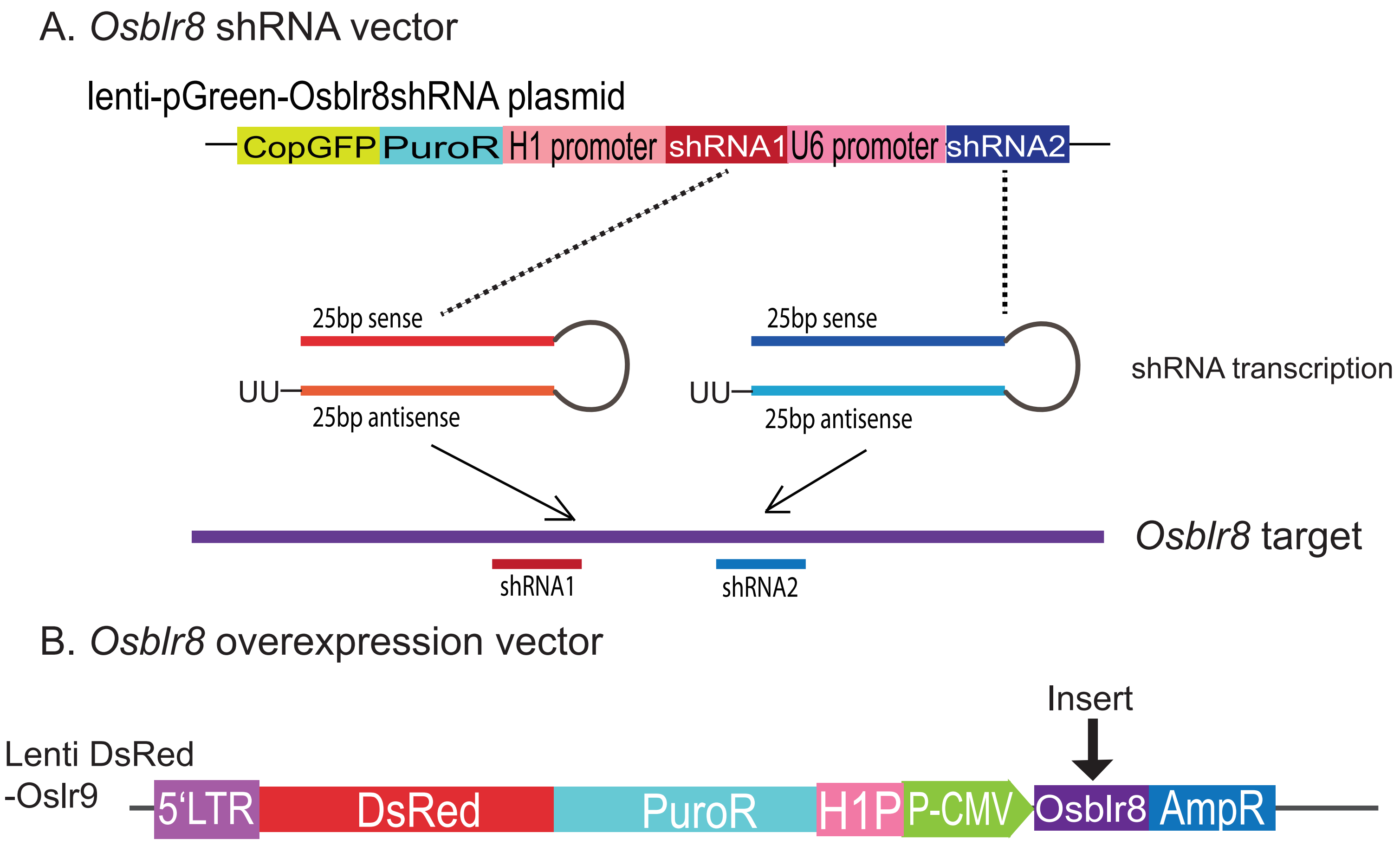

Figure S2. *Osblr8* knockdown and overexpression

A. Identification *Oct4/Sox2* promoter binding lncRNA *Osblr8* by CRIST-seq

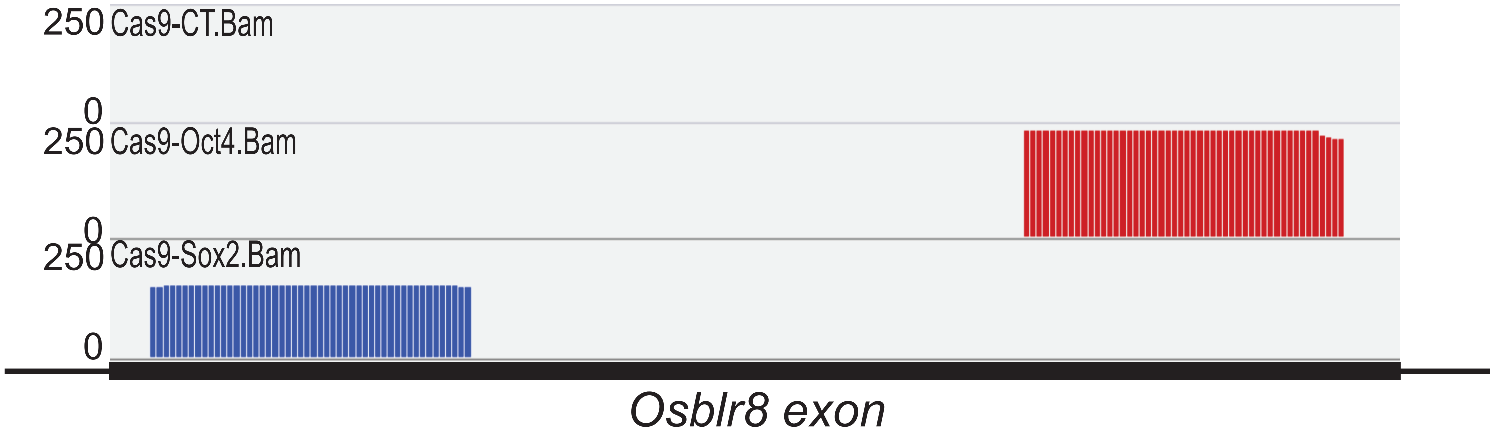

Figure S3. CRIST-seq identifies the *Osblr8-Oct4-Sox2* interaction

SUPPLEMENTAL TABLES

Table S1. Oligonucleotide primers used for PCR

ID	Oligo Name	Oligo sequence	Product size
RT-PCR			
Osblr8	JH5158	CAGCCTGAAACCTGCTTGCT	131bp
	JH5159	GACCCGATTCCCGCCGAAT	
Oct4	JH116	CAATGCCGTGAAGTTGGAGAAG	179bp
	JH117	GGCTGAACACCTTTCCAAAGAGA	
Sox2	JH118	GGTTACCTCTTCCTCCCACTCCAG	193bp
	JH119	TCACATGTGCGACAGGGGCAG	
Nanog	JH120	TCTCCTCCATTCTGAACCTGAGC	150bp
	JH121	TGCTGGGATACTCCACTGGTGCT	
β-Actin	J880	CAGGTCATCACCATTGGCAATGAGC	135bp
	J881	CGGATGTCCACGTCACACTTCATGA	
TET1	JH6031	GAACAGCCAYCAGATCTGTAAG	170bp
	JH6032	CTGAYTTGGGGCCATTTACTG	
TET2	JH6033	GTCCTYATGTGGCAGCTATTAG	129bp
	JH6034	TAGCAATAGGACATCCCTGAG	
TET3	JH6035	CYAAGAGTCTGCTGGACACAC	154bp
	JH6036	TCCTCCATGAGTTCCCGGATA	
RAT Oct4 binding			
5’-CT	JH4348	CTGAGTCCTCTGCAAGATGC	137bp

	JH4349	CCAAGGCACCTGCCTAGGATT	
P1	JH4352	AGTTGTCCCCAGGGGAGCCAT	140bp
	JH4353	AAGGGGCCTGGGAGGGACTG	
P2	J648	CAGAGGATGGCTGAGTGGGCTGTA	123bp
	JH4354	CACCCCTGCCTTGGGTCACCG	
5'-Enh	JH4350	CAGATGAGCCAACAGGTCTG	125bp
	JH4351	CAGCAACTTTGTCTGAAGTCC	
3'-Enh	JH4407	GTGGAGCAGGCGAAACTTGC	123bp
	JH4408	ATTCCATCGGCAGCCTCAGC	
Exon1	JH4664	TCAGGTTGGACTGGGCCTAG	121bp
	JH4665	GCGGTCGGCACAGGGCTCAG	
Exon4	JH4668	GCAAATCGGAGACCCTGGTGCA	110bp
	JH4669	TAGGGAGGGCTTCGGGCACT	
3'-CT	JH4357	CGCCCTACTCTGATGTTCGA	121bp
	JH4358	TGAGCCATCTCTCCAGCATC	
Osblr8 shRNAs			
1		CTGGAACCTGAGGAGCCACACACGT	
2		TGCACCTTTCTACTGGACCAGAGAT	
shCT		GCAGCAACTGGACACGTGATCTTA	
Osblr8 overexpression primers			
JH6195	TCGGGCGCCAGATATCTGTAGTCTCCCCTCCCCCAGCCT		
JH6196	CAGAATCGAAGAATTCGTTACTCTGATCAAGGCTCAAATT		
CRIST Cas9 gRNA			
Oct4-1		GAACATTCAATGGATGTTTT	

Oct4-2	GTGTGAGGGGATTGGGGCTC
Sox2-1	GGGGTTGAGGACACGTGCTG
Sox2-2	GAGCCAATATTCCGTAGCAT
Control (gCT)	GAAGTGGGATGATCCTCTGA
RAT primers	
Osblr8	
JH5159	GACCCGATTCCCGCCGAAT
JH6193	TTCCAGCAGTGGGCGTGGCA
JH6194	GTTACTCTGATCAAGGCTCAAAT
RAT control	
JH5849	ATGGACTGATGATCTTATGC
JH5850	TACATAGTAGATCAGATACT
3C primers	
Oct4 functional loop	
P780	ACCATCTCTGGCTGGGGACGTG
P783	GCAGACAGGCACTCTGAGGGC
P785	GGACACCTGGCTTCAGACTTCG
P790	ACTGACTGCTCTGCCAGAGGTC
Ercc3 ligation control	
JH6286	TGGAGCAGTGGAAAGCCCAGT
JH6282	CTTCATAAGTGTCTTAGCAGAGCT

Supplement Figure Legends

Figure S1. *Osblr8* is highly expressed in pluripotent stem cells

A. *Osblr8* is located in chromosome 3. The total length of *Osblr8* is 210 bp. Differential expression of *Osblr8* in iPSCs and fibroblasts (RNA-seq IGV). The expression value between iPSC and fibroblasts of *Osblr8* is 4164 vs 34.

Figure S2. *Osblr8* knockdown and overexpression

A. *Osblr8* shRNA vector: Two shRNAs were designed from two sites of *Osblr8* lncRNA. shRNAs were cloned into one lentiviral plasmid vector and were controlled by H1 and U6 promoters, respectively. U6: RNA polymerase III U6 promoter; H1: human H1 RNA polymerase III promoter; PuroR: puromycin resistance. CopGFP: Green Fluorescent Protein reporter.
B. *Osblr8* overexpression vector. *Osblr8* was cloned in a lentiviral vector and was controlled by CMV promoter. DsRed: the fluorescent marker gene; PuroR: puromycin selection marker.

Figure S3. CRIST-seq identifies the *Osblr8-Oct4-Sox2* interaction.

A. The *Osblr8-Oct4-Sox2* interaction by CRIST-seq. The CRIST-seq data was analyzed by IGV software. The *Oct4* and *Sox2* promoter binding value are 253 and 167, respectively. The Cas9 random (gCT) CRSIT-seq control did not show any interaction signals with *Osblr8* lncRNA. The predominant binding sites for *Oct4* and *Sox2* are two 50 bp elements.
